# A case report and review of rheumatoid arthritis co-occurring with tuberous sclerosis complex, a rare occurrence

**DOI:** 10.3389/fimmu.2024.1425988

**Published:** 2024-09-26

**Authors:** Hai-Qin Yin, Xue-Fei Li, Yao Fu, Hui-Ling Zhu, Yu-sheng Luo

**Affiliations:** Department of Rheumatology and Immunology, Jiujiang University Affiliated Hospital, Jiujiang, Jiangxi, China

**Keywords:** pulmonary lymphangioleiomyomatosis, angiomyolipoma, periungual fibroma, rheumatoid arthritis, tuberous sclerosis

## Abstract

Rheumatoid arthritis (RA) is a common autoimmune disease. Tuberous sclerosis complex(TSC) is a rare autosomal dominant disorder. We report a case of RA with TSC. The patient was a 46-year-old woman with polyarthritis and cough symptoms, rheumatoid arthritis associated interstitial lung disease (RA-ILD) was initially considered, and after more than 3 months of anti-rheumatic treatment, the patient still had cough, and further examination revealed that the patient had lymphangioleiomyomatosis in the lungs, hepatic and renal angiomyolipomas, multiple subependymal nodules, Vertebral osteosclerotic nodules, as well as facial angiofibromas and periungual fibroma, RA was finally diagnosed with TSC, and everolimus 10mg qd was added to anti-rheumatic therapy for 1 month, and the patient’s cough symptoms were relieved.

## Introduction

The main manifestations of rheumatoid arthritis are symmetrical, invasive facet joint swelling and pain, and the basic pathological feature is synovitis, which often involves multiple joints, and gradually develops bone and joint destruction with the progression of the disease, resulting in a high disability rate. Extra-articular lesions can cause cardiovascular, respiratory, and hematologic damage. The global incidence of RA is about 1%, with two-thirds of women occurring. Tuberous sclerosis, also known as Bourneville’s disease, is inherited in an autosomal dominant manner and is caused by overactivation of the mammalian target of rapamycin complex 1 (mTORC1) pathway due to mutations in the TSC1 or TSC2 genes. The TSC1 gene is located on chromosome 9q24 and encodes the hamartin protein, and the TSC2 gene is located on chromosome 16p13 and encodes the potato protein tuberin. These two proteins together with TBC1 domain family member 7 (TBC1D7) protein form a TSC complex, negatively regulate the biological target Rheb protein of mTORC1, regulate cell cycle, apoptosis, autophagy and cytoskeletal reorganization, and lead to the formation of hamartomas in multiple organs, which can affect the skin, brain, kidney, lung, oral cavity and other organs in the body.TSC2 mutations are more common than TSC1 mutations, and TSC2 mutations often lead to more severe clinical manifestations. The vast majority of TSC1 variants are nonsense and frameshift variants, and the most common TSC2 variants are missense variants, followed by deletion or recombination of large fragments. The incidence of TSC is 1 in 6,000 to 10,000, and there are about 2 million people worldwide. Clinically, TSC is a rare disease, and there are no reports of RA complicated with TSC and everolimus in the effective treatment of two diseases.

## Case report

**Figure 1:**
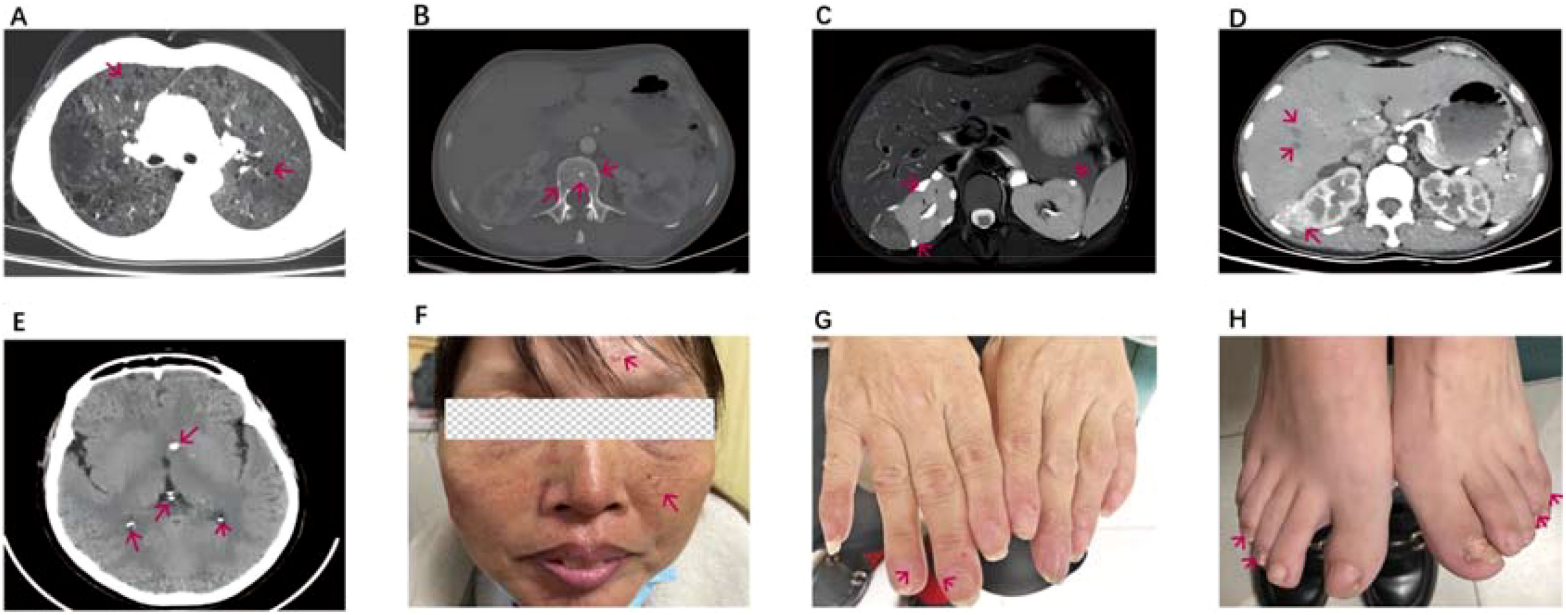
**(A)** CT scan of the chest shows sac -like translucent and ground-glass opacities in both lungs **(B)** CT of the spine shows multiple high-density nodules in the vertebrae. **(C)** MRI enhancement shows abnormal enhancement of multiple nodules in the liver and bilateral kidneys. **(D)** CT enhancement showed very low-density opacities in the liver and kidney, as well as intrarenal mass opacities. **(E)** CT brain shows bilateral paraventricular nodular hyperdensity. **(F)** a few hemangiomatous rash on the patient's face and fibrous plaques on the forehead. **(G)** Fibroids on the fingers **(H)** Fibroids on the toes. The red arrows indicate the abnormalities observed on imaging and the patient's abnormal signs.

**Figure 2:**
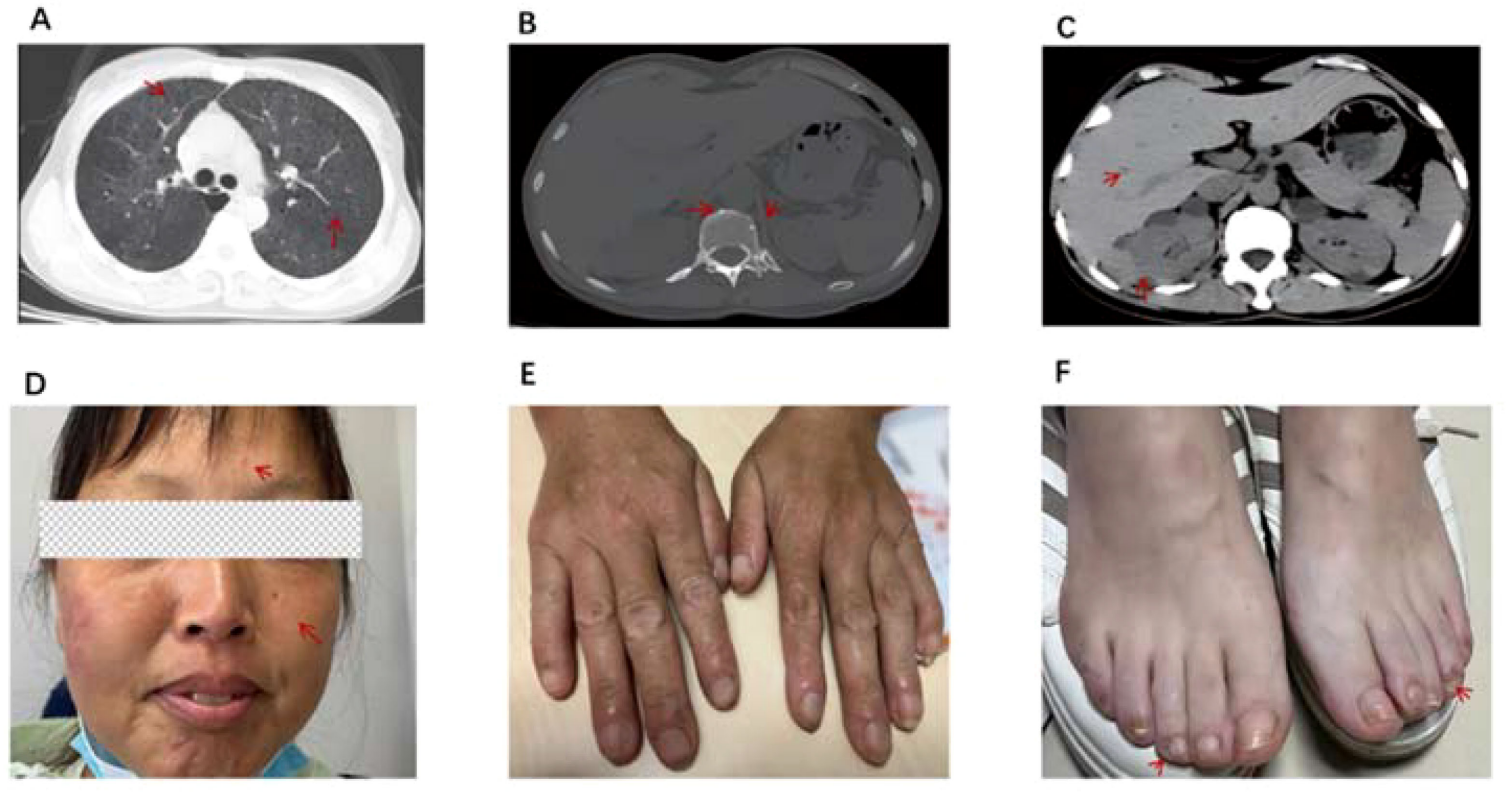
**(A)** CT scan of the chest shows pulmonary cystic degeneration is the same as before, however, ground-glass changes in the lungs are less severe than before. **(B)** CT of the spine shows vertebral osteosclerotic nodules remain. **(C)** CT scan of the abdomen showed multiple nodules in the liver and multiple high-density nodules in the kidneys. **(D)** Fibroids on the forehead and face are smaller than before. **(E, F)** Periungual fibroids are partially reduced. The red arrows indicate the abnormalities observed on imaging and the patient's abnormal signs after treatment.

The patient is a 46-year-old female. Recurrent multi-joint swelling and pain for more than 3 years with cough for 1 year, and she was presented in June 2023. No history of smoking, no pneumoconiosis, no history of epilepsy. The patient did not have standardized treatment within 3 years of the onset of arthritis, and lung imaging showed cystic translucent opacity and ground-glass changes, and ILD was considered, and no standardized treatment was performed. At the time of hospitalization, she presented with swelling and tenderness in both wrist joints, metacarpophalangeal joints of both hands, proximal interphalangeal joints, shoulder joints, and knee joints. Laboratory tests suggest rheumatoid factor 155.40 IU/ML and erythrocyte sedimentation rate 46 MM/H. C-reactive protein 37mg/L, anti-CCP antibody 185.9U/ml, IgG 17.6g/L,ANA and ANCA negative, PCT, T-SPOT, HIV, syphilis-related tests were normal, blood cell analysis, liver and kidney function and tumor markers were normal. The DAS28 (ESR) score was 7.46 and the DAS28 (CRP) score was 7.05, both of which were in high disease activity. Noncontrast magnetic resonance imaging of the hand suggests synovitis of the wrist joint, multiple bone marrow edema. Chest CT scan showed that the two lungs were scattered with cystic translucent opacities and ground-glass opacities. Initial consideration of rheumatoid arthritis, interstitial pneumonia. After 3 months of anti-rheumatic treatment with prednisone 30 mg once a day(Tapere), leflunomide 20 mg once a day, and hydroxychloroquine 0.2 g twice a day, the patient’s cough was not significantly relieved, and the patient’s joint swelling and pain recurred during the prednisone reduction process. Chest CT again and whole abdominal CT + enhancement showed that the two lungs were scattered with cystic translucent opacities and ground-glass opacities (**Figure 1A**). multiple high-density nodules in the vertebral bones (**Figure 1B**); Renal magnetic resonance scan + contrast examination showed liver and kidney angiomyolipoma (**Figure 1C**). CT enhancement showed very low-density opacities in the liver and kidney, multiple cysts in the liver and kidneys, liver hamartomas, mass and nodular shadows in both kidneys (**Figure 1D**). Cysts in both kidneys. CT of the head showed bilateral paraventricular nodular hyperdense opacities (**Figure 1E**). Further examination reveals a few hemangiomatous rash on the patient’s face (**Figure 1F**), Fibroids on the fingers (**Figure 1G**) and the toes (**Figure 1H**), and a history is followed up that the patient’s father and niece also have facial fibroids. The patient and his family did not pay attention to this facial lesion in the past, and no examination and treatment were performed. This time, it is necessary to determine whether there is a gene mutation. Tuberous sclerosis TCS1 and TSC2 gene testing was performed: no single nucleotide sequence variations, short insertion variants and copy number variations related to clinical phenotype were detected. In the end, RA was diagnosed with TSC, and prednisone 10 mg qd, leflunomide 20 mg qd, hydroxychloroquine 0.2 bid, and everolimus 10 mg qd were treated for 1 month, and the patient’s joint swelling, pain and cough symptoms improved, and the arthritis symptoms of the patient continued to be relieved after 6 months of follow-up, erythrocyte sedimentation rate 20 MM/H; C-reactive protein 8.4mg/L,IgG was 11.52g/L, and the laboratory tests showed that the disease was in gradual remission, with a DAS28 (ESR) score of 2.1 and a DAS28 (CRP) score of 1.77. CT scans of the lungs and kidneys showed no significant progress in the cystic changes of the lungs and kidneys, and vertebral osteosclerotic nodules still existed, but the ground-glass changes in the lungs were slightly reduced compared with before, the facial fibromatous lesions were reduced compared with before, and the periungual fibroids were partially reduced (**Figure 2**). Unfortunately, We did not have post-treatment magnetic resonance imaging at the follow-up. During the follow-up process, the patient had menstrual disorders, mainly manifested in prolonged menstrual periods, and under the premise of excluding gynecological diseases, considering the adverse reactions of everolimus, the dose of everolimus was reduced to 5mg qd, prednisone was reduced to 5mg qd, leflunomide 10mg qd and hydroxychloroquine 0.2g qd, and the patient’s menstrual disorders disappeared.

## Discussion

In this case, 18 facet joints and 4 large joints were swollen and tender, blood tests showed positive rheumatoid factor and high titers of anti-CCP antibodies, ESR and CRP are elevated, and imaging of the diseased joints showed synovitis, which met the RA 2010 ACR/EULAR classification criteria. Due to the imaging manifestations of the lungs with round thin-walled cysts with different morphologies, RA-ILD was considered at the initial stage, and TSC was diagnosed according to the 2021 International Clinical Diagnostic Criteria for Tuberous Sclerosis, combined with facial angiofibromas and fibrous plaques of the forehead, periungual fibromas, subependymal multiple nodules, pulmonary lymphangiomyomas, hepatic and bilateral renal angiomyolipomas, multiple renal cysts, and vertebral osteosclerotic nodules. The clinical manifestations of TSC are complex and one of the few genetic disorders that can be diagnosed based on clinical manifestations alone.

The coexistence of RA and TSC has been reported very rarely. HLA-DR4 is associated with the pathogenesis of RA, while the pathogenesis of TSC is related to the tumor suppressor genes TSC1 or TSC2 (1). In some sporadic cases, mosaic mutations or no genetic mutations may be present. The pathogenesis of RA is not fully understood. Studies have shown that the mammalian target of rapamycin (mTOR) signaling pathway is involved in the development of RA disease, and the abnormality of this pathway can recruit and activate immune cells and fibroblast-like synovial cells (FLS) at the arthritis site, resulting in the production of a variety of chemokines, pro-inflammatory cytokines, and cathepsin, degrading the extracellular matrix and cartilage, and causing persistent inflammation and tissue damage (2, 3). The mTOR signal pathway is very complex and can be divided into two signaling pathways: LKB1/AMPK/TSC/mTOR and PI3K/Akt/TSC/mTOR. The main function of the LKBl/AMPK/TSC/mTOR pathway is to activate AMP-activated protein kinase (AMPK) to rapidly initiate negative regulatory mechanisms, inhibit lipid synthesis and reduce energy expenditure through activated AMPK. Aberrant signaling of this pathway can lead to enhanced glycolysis, which in turn exacerbates metabolic disorders and leads to the development of autoimmune diseases. The PI3K/Akt/TSC/mTOR signaling pathway is involved in B cell activation and plays an important role in osteoclast differentiation and survival, and studies have demonstrated that mTOR activity is highly activated in synovial tissue, especially synovial osteoclasts, in patients with rheumatoid arthritis (4, 5), and inhibition of this pathway can prevent bone destruction in rheumatoid arthritis. The mTOR signaling pathway can also participate in the occurrence of immune diseases by changing the level of autophagy and oxidative stress. mTOR activity is increased in a variety of connective tissue diseases (6).

At the same time, the mTOR signaling pathway is considered to be the key to the development of TSC disease, and is involved in the process of various clinical manifestations of TSC: SEGA, renal AML, LAM, facial fibroids and other lesions. Henske EP et al.[7, 8] mentioned that the mTOR signaling pathway was activated in LAM, and mTOR overexpression occurred, resulting in abnormal proliferation of LAM cells. Numerous studies have also shown that (9, 10) loss of Tsc1 in proximal tubular epithelial cells induces mTORC1 activation, which can induce cell proliferation and renal fibrosis. Hongdi Cao et al. (11) found that mTORC1 signaling can also mediate the progression of renal interstitial fibrosis by regulating glycolysis of proximal tubular epithelial cells, and inhibition with rapamycin can inhibit renal tubular epithelial cell proliferation and block TGF-b1-induced tubular epithelial to mesenchymal transition. In the neurological manifestations associated with TSC, Paola Zordan et al. (12) found that the tumor progression from static subependymal nodules (SENs) to subependymal giant cell astrocytomas (SEGA) was accompanied by mTORC1 overactivation in a mouse model of TSC, and that sustained activation of Akt and mTORC2 in neural stem cells was a necessary step to induce SEN and SEGA.

There are few clinical reports of immune diseases complicated with TSC. Only one case of Sjögren’s syndrome complicated with pulmonary lymphangioleiomyoma (LAM) was found (13). We reported this case of RA merging TSC, in addition to the currently known pathogenic factors, we reported that the overexpression of mTOR may occur due to the activation of mTOR, resulting in the recurrence of joint synovitis and the abnormal proliferation of LAM cells leading to multiple cystic changes in the lungs.

At present, TSC cannot be completely cured, and symptomatic treatment is the mainstay. The mTOR inhibitors sirolimus and everolimus have been recognized by experts at home and abroad as effective drugs for the treatment of TSC (14). Masaki Hirose et al. (8, 15) showed that treatment with sirolimus reduced serum VEGF-D levels, a biomarker for the diagnosis and treatment of LAM, and stabilized lung function in LAM patients. In addition, Everolimus is also widely used in the treatment of SEGA and AML (16, 17). mTORC1 inhibitors have been shown to be effective in reducing the size of angiomyolipomas (18). Studies have confirmed that the response to treatment of iveolimus is equivalent regardless of the patient’s TSC1 or TSC2 mutation status (19). One randomized, double-blind controlled study suggests that everolimus significantly reduces the frequency of TSC-related refractory seizures (20).

There are also data to suggest that sirolimus can be used to treat immune diseases such as RA, effectively alleviate arthritis symptoms and restore Th17/Treg cell balance during treatment (21, 22).

In this case, the patient developed menstrual irregularities during the treatment. The main drugs used in the treatment of patients were leflunomide, hydroxychloroquine, and everolimus. According to the instructions for leflunomide, the black box warning of the United States FDA for leflunomide is embryotoxicity and hepatotoxicity, and other common adverse reactions include diarrhea, itching, hair loss, rash, etc., and the reproductive toxicity mechanism may be related to its inhibition of dihydroorotate dehydrogenase activity; Hydroxychloroquine is less gonadal toxic, the mTOR inhibitor: everolimus can reduce progesterone levels in women and testosterone levels in men, which in turn can lead to menstrual disorders in women. A randomized, double-blind, placebo-controlled phase III trial, EXIST-2, reported multiple adverse effects associated with everolimus, including menstrual disorders (menorrhagia) accounting for 8% and vaginal bleeding unrelated to normal menstruation (7%) (23), In the EXIST-2 extension study, 31% of patients in United States experienced at least one episode of amenorrhea. Studies have also shown that 23.35%~65% of female patients experienced menstruation-related adverse reactions, and some patients needed to receive hormone therapy (24, 25). Therefore, it is considered that the heavy menstrual flow and prolonged menstrual period in this patient are closely related to everolimus, and the menstrual disorders of the patients disappeared after low-dose use of everolimus. At present, a number of studies at home and abroad have shown that low-dose mTOR inhibitors can effectively control the disease, reduce the possibility of adverse events with long-term use of everolimus, reduce the economic burden of patients, and bring better tolerability and economy.

## Conclusion

In this case, the patient was treated with leflunomide, hydroxychloroquine, and hormone immunosuppressive therapy, but still had recurrent cough and joint swelling and pain, and was treated with mTOR inhibitor: everolimus, and the 6-month follow-up showed that the patient’s joint inflammation and cough symptoms continued to be relieved, and there was no obvious progress or deterioration in the imaging of the lungs and kidneys. mTOR inhibitors can induce autoimmune tolerance to reduce joint inflammation and are expected to be a new option for the treatment of RA.

## Data Availability

The datasets presented in this study can be found in online repositories. The names of the repository/repositories and accession number(s) can be found in the article/supplementary material.
